# Characterization of Chia Seeds, Cold-Pressed Oil, and Defatted Cake: An Ancient Grain for Modern Food Production

**DOI:** 10.3390/molecules28020723

**Published:** 2023-01-11

**Authors:** Diana Melo Ferreira, Maria Antónia Nunes, Liliana Espírito Santo, Susana Machado, Anabela S. G. Costa, Manuel Álvarez-Ortí, José E. Pardo, Maria Beatriz P. P. Oliveira, Rita C. Alves

**Affiliations:** 1 LAQV/REQUIMTE, Department of Chemical Sciences, Faculty of Pharmacy, University of Porto, Street of Jorge Viterbo Ferreira, 4050-313 Porto, Portugal; 2 Higher Technical School of Agricultural and Forestry Engineering, University of Castilla-La Mancha, Campus Universitario, s/n, 02071 Albacete, Spain

**Keywords:** amino acids, cold-pressing, fatty acids, food security, nutritional analysis, vitamin E

## Abstract

The increasing demand for superfoods has resulted in an increase in chia seeds consumption. The reintroduction of this ancient crop in agriculture is useful to ensure food security since it can grow in high-stress conditions. The current study aimed to characterize chia seeds, cold-pressed oil, and defatted cake (the oil extraction by-product) to improve their value and to meet consumer’s expectations (low-fat products). Chia seeds presented a significantly higher energy value than cake (444 vs. 284 kcal/100 g, respectively) due to fat removal (33 vs. 7%). The cake showed higher contents of total minerals (6 vs. 5%), protein (27 vs. 18%), and fiber (48 vs. 38%) in comparison to the seeds, and was hence considered a promising food ingredient. The major fatty acid in oil, seeds, and cake was α-linolenic acid (62–66%), and the vitamin E content was 409, 200, and 44 mg/kg, respectively. The major amino acid in the seeds and cake was glutamic acid (49 vs. 36 mg/g). The oil had a low oxidative stability (1 h), and the total phenolics content was 1.3 mg gallic acid equivalents/100 g. Chia cake incorporation in food formulations will follow consumer’s interests, and the obtained oil can be used to improve the oil supply for different applications. This approach adds value to the concept of “one health” since it includes the culture, the environment, and the consumers.

## 1. Introduction

Chia seeds (*Salvia hispanica* L.) are an ancient grain from Mexico, Colombia, and Guatemala. In 2013, they were labelled as a “novel food” (Regulation (EC) No 258/97 of the European Parliament), and in 2017, chia oil was considered safe for human consumption in the United States Pharmacopeia. These seeds are considered a functional food since their consumption has been correlated with lower triglyceride and cholesterol levels as well as lowering blood pressure and decreasing the risk of cardiovascular diseases; the seeds also have antidiabetic and hepatoprotective activities and provide protection against arthritis, autoimmune diseases, and cancer [1]. All these properties allow us to consider chia seeds as a superfood.

The term superfood does not have an official definition, but it is used to refer to foods that can provide nutrients in high quantities in diets and contribute to the proper functioning of the body [2]. Chia seeds belong to the group of nuts and seeds that also includes flaxseeds, hemp seeds, and carob seeds [2]. When comparing the four, carob seeds present the lowest energy value, followed by chia seeds, while flax and hemp seeds present similar values (346, 486, 534, and 553, respectively). These values can be explained by the higher fat contents in the last two (around 42 and 48%, respectively), whereas carob seeds only have 2% of fat. Nevertheless, most acids in chia, flax, and hemp seeds are polyunsaturated acids, which have a beneficial health impact. Among the four, chia seeds have the highest fiber content (34%). The protein content in hemp seeds is 32% while that of chia is only half that value. All the seeds have high levels of important minerals. The levels of P, Na, K, Ca, Mg, Fe, and Zn in chia seeds are 860, 16, 407, 631, 335, 7.72, and 4.58 mg/100 g, respectively. Chia seeds also have high levels of vitamins of the B complex and vitamins C and E as well as carotenoids and phenolic compounds [2]. Furthermore, the versatility of chia seeds in cooking is enormous, especially due to their ability to form a gel that provides thickening, emulsifying, and stabilizing properties; this ability is a result of the high fiber content [1]. The reported benefits, neutral taste, and convenience for incorporation in foodstuffs raised its popularity and consequently its increased demand for consumption [3].

The chia seed market is expected to grow by 6.5% each year between 2022–2027. The fastest growing market is Europe, mostly because of consumers’ search for superfoods, wholesome and natural ingredients, and the adoption of vegetarian food patterns. North America is the current largest consumer, while Latin America (Paraguay, Bolivia, Argentina, and Mexico) is the leading producer of chia seeds worldwide [3]. 

The increase in the cultivation of this plant might also result in the improvement of land degradation and soil erosion. In the sense that it can benefit agriculture since it can grow in semi-arid regions, contributing to food security [3,4].

Cold-pressing is an oil extraction method that preserves natural antioxidants. The recent trends for the use of natural products also motivated the consumption of cold-pressed oils. As they are considered specialty oils with high market prices, adulteration with lower-quality oils is now a common concern [5]. Accordingly, in the current study, a global chemical characterization of cold-pressed chia oil was performed, aiming to assess the nutritional profile and discuss quality control, food applications, and health-promoting traits.

The processing of oilseeds produces large amounts of by-products, regarded as a residue/waste but they are an added-value biomass [6]. Chia cake (an oil extraction by-product) was analyzed as it deserves valorization as an ingredient for sustainable diets and food production. An overall chemical analysis of chia seeds was also performed to valorize this grain since it is a novel food with increasing popularity. An evaluation of the protein quality of chia cake and seeds was also performed. The main objective of this study was to clarify the advantages of chia seeds consumption (health impact), the environmental importance (growing in arid conditions), and finally, the contribution to increase food availability (adding value to the cake).

## 2. Results and Discussion

### 2.1. Cold-Pressed Chia Oil

The chemical analysis of cold-pressed chia oil is presented in Table 1. Cold-pressed oils are considered specialty oils due to high market prices [5]. The content of total vitamin E (409 mg/kg) and the total phenolic compounds—TPC (1.3 mg gallic acid equivalents—GAE/100 g) might contribute to chia oil stability. This oil is very rich in polyunsaturated fatty acids (85% PUFA), which are more prone to oxidation than monounsaturated fatty acids (6% MUFA) and saturated fatty acids (9% SFA). Accordingly, a low oxidative stability (1 h) was determined. Ixtaina et al., 2012, reported a higher induction time (2.3 h) for a higher amount of oil (5 g) at a lower temperature (98 °C) [7]. The same happened in a study conducted by Bordón, in 2019 (2.57 h, 5 g, 100 °C) [8]. The low oxidative stability obtained in the present work (3 g, 120 °C) agrees with the total flavonoids content—TFC that was not detected in chia oil nor in the 2,2-diphenyl-1-picrylhydrazyl radical—DPPH^•^ inhibition assay. Furthermore, the ferric reducing antioxidant power—FRAP result was low (35 μmol ferrous sulphate equivalents—FSE/100 g).

Vitamin E is a combination of 8 liposoluble compounds, but in chia oil, only α-, γ- and δ-tocopherols were identified in different amounts (5, 389, and 15 mg/kg, respectively). A similar profile was previously reported by Ciftci et al. in 2012 (8, 422, and 15 mg/kg, respectively, total value: 445 mg/kg) [11]. Ixtaina et al., in 2011, reported total contents of α-, γ-, and δ-tocopherols varying between 238–427 mg/kg; the results obtained in the present study are within this range [12]. Oliveira-Alves et al., in 2017, reported a slightly higher TPC (2 mg GAE/100 g) in comparison to the present data, but an ultrasound-assisted extraction was performed, improving the rupture of cell walls [10]. In the previous study, on a FRAP (ferric reducing antioxidant power) assay, a result of 26 µmol Trolox equivalents—TE/100 g was obtained but a different standard was used (Trolox), impairing direct comparison with the results of the present study [10].

The profile of fatty acids (FA) (Table 2) revealed high quantities of α-linolenic acid (n3, ALA, 66%), followed by linoleic acid (n6, LA, 19%), and a very low n6/n3 ratio (0.3). This is beneficial for health since it counterbalances the European diet that is richer in n6 FA and SFA [1], but it results in low-stability oils as shown on the oxidative stability test. Unlike the results obtained in the present work, other FA were reported in another study, e.g., C14:0, C15:0, C17:0, C17:1, C20:0, C20:1n9, and C22:0, although in low percentages (0.03–0.29%) [13]. Since the analyses were performed immediately after oil extraction, no products of primary or secondary oxidation were formed (K_232nm_ and K_270nm_, respectively, Table 1). Moreover, the peroxide value was low (2.4). The latter value was within the interval of values (0.14–2.67) reported by Martínez et al., in 2012 [9].

Previous results did not show high concentrations of the TPC or TFC in antioxidant activity assays. The oil is protected inside the seed; thus, it is not exposed to environmental stresses. Therefore, these parameters were also evaluated in the seeds and cake (results discussed below) since the hull is likely to have more of these compounds as it is in direct contact with the environment, for example, oxygen attack.

Nevertheless, this oil can have further applications as a dietary supplement [1] or it can be used to produce oil blends with other oils richer in MUFA and SFA, which will increase its oxidative stability, similar to almond or sesame seeds as proposed by other authors [8,16]. In particular, sesame seed oil is richer in MUFA (42.9%), mostly oleic acid (42.7%), and this FA is known to confer higher stability to food products [17].

### 2.2. Chia Seeds and Cake

The macro composition of chia seeds and cake (Table 3) revealed that the seeds presented a significantly higher energy value than the cake (444 vs. 284 kcal/100 g, respectively) due to the expected fat removal (33 vs. 7%, respectively) by cold-pressing. This method was effective in removing most of the oil. Consequently, the cake, compared to the seeds, presented significantly higher contents of ash (6 vs. 5%, respectively), total protein (27 vs. 18%, respectively), total dietary fiber (48 vs. 38%, respectively), insoluble fiber (39 vs. 35%, respectively), and soluble fiber (9 vs. 3%, respectively). The present results for chia seeds (Table 3) are in agreement with the intervals reported by da Silva et al. in 2017 [18], particularly, moisture content (6.6%: 5.6–7.1%); ash content (4.7%: 4.6–5.1%); total protein content (18%: 18.2–19.7%); total fat content (32.9%: 30.2–32.2%); and total dietary fiber (37.7%: 33.4–37.2%). The total dietary fiber in chia seeds (37.7%) was also in agreement with that reported by Scapin et al. in 2016 (37.4%) [19].

The high contents of dietary fiber in chia seeds may confer water-holding and oil-holding capacity to food formulations. In contact with water, chia seeds form a polysaccharide gel with emulsifying properties, thereby functioning as a stabilizing, texturizing, and thickening agent; this is very useful in the food processing industry for developing new food products [1]. The high fiber content may also benefit the gut microbiota, with benefits for the host metabolism. For example, the microbial enzymes complement the activity of digestive enzymes present in the gut and liver to produce metabolites like short-chain fatty acids and peptides; participate in the reabsorption of bile salts back to the liver; aid in the digestion of some food components; participate in cellular signaling and genes expression modulation; and protect against harmful bacteria via competition and secretion of antimicrobial components [22,23].

Regarding the content of protein, the cake had a significantly higher content of total amino acids—AAs (269 vs. 203 mg/g, Table 3). The major AAs were the same in both the seeds and cake samples—glutamic acid, followed by arginine and aspartic acid—but chia cake presented the highest amounts (49 vs. 36, 34 vs. 26, and 24 vs. 18 mg/g, respectively, *p* < 0.05). Both samples were dietary sources of all essential AAs (96 vs. 74 mg/g, *p* < 0.05). Particularly, branched-chain AAs (leucine, isoleucine, and valine) were identified in both samples (18 vs. 14 mg/g for leucine, 10 vs. 7 mg/g for isoleucine, and 12 vs. 9 mg/g for valine.

The protein quality of chia seeds and cake is presented in Table 4. The limiting amino acid in the chia seeds was valine, since it presented the lowest amino acid chemical score (AACS, 125%). However, in the cake, it was methionine (AACS: 79%). The essential amino acid indexes were high for both samples (EAAI, cake: 106%; seeds: 128%). Overall, these samples presented a balanced AA composition, since they contained all the essential AAs for adults, with high values of glutamic acid, and the EAAIs were very high (>90%) [24]. A similar profile was previously obtained for sesame seeds and defatted flour [17]. Similar to sesame seeds, chia seeds are a suitable plant-based alternative for people with vegetarian food habits. Furthermore, chia seeds are gluten-free, so it can be consumed by patients with coeliac disease and people who avoid gluten in their diets. The incorporation of chia protein, especially its cake, can nutritionally enrich various foodstuffs, providing high-protein levels. This cake is also a gluten-free alternative to wheat flour.

The FA profile of chia seeds and cake (Table 2) revealed a high content of α-linolenic acid (ALA, C18:3n3c, 66 vs. 62%, respectively, *p* < 0.05), followed by linoleic acid (LA, C18:2n6c, 19 vs. 20%, respectively, *p* < 0.05), and palmitic acid (C16:0, 8 vs. 7%, respectively, *p* < 0.05), with high PUFA contents (85 vs. 82%, respectively, *p* < 0.05), low n6/n3 ratios (0.29 vs. 0.33, respectively, *p* < 0.05), and low n9/n6 ratios (0.29 and 0.28, respectively). Unlike the results obtained in the current study, the results in previous studies showed other FA in chia seeds but in low percentages (0.03–0.16%): C14:0, C15:0, C17:1, C20:1n9, C20:2, and C22:0 [11,14]. 

Since chia samples have high contents of PUFA (seeds: 85; cake: 82%), assessing the presence of antioxidants that can protect it from oxidation seems to be a relevant approach. Chia seeds presented significantly higher contents of vitamin E than the cake (Table 3), with a total value of 200 vs. 44 mg/kg, due to the higher amount of fat in the seeds and liposolubility of vitamin E. In terms of the vitamin E profile, from the 8 isomers, only α-, γ-, and δ-tocopherols were identified in both samples (59 vs. 14, 137 vs. 29, and 3 vs. 1 mg/kg, respectively) in accordance with the profile reported for the oil.

Like vitamin E, phenolic compounds help to maintain the quality and nutritional value of foods by preventing or delaying deterioration. They are also able to protect body tissues from oxidative damage. The antioxidant mechanism of vitamin E involves the elimination of free radicals or the decomposition of primary oxidation products to non-radical compounds [26]. There were no significant differences in the TPC (Table 3) between chia cake and seeds (199 and 188 mg GAE/100 g fresh weight—FW, respectively) nor in the results of the DPPH^•^ inhibition assay (144 and 130 mg TE/100 g FW, respectively). However, the TFC was significantly higher in the cake than in the seeds (309 vs. 221 mg ECE/100 g FW, respectively), as well as in the FRAP assay (15 vs. 11 mmol FSE/100 g FW, respectively). Scapin et al., in 2016, obtained chia seed extracts with 80% aqueous ethanol stirred at 60 °C (different experimental conditions in comparison to the present study) and reported a TPC of 2.6 g GAE/kg, a TFC of 0.16 g quercetin equivalents/kg, and an FRAP assay result of 45 mmol TE/kg in dry weight—DW [19]. Saphier et al., in 2017, also used a different extraction solvent (50% aqueous ethanol) and obtained a TPC of 199 mg GAE/100 g DW [27]. In another study, the results of the FRAP and DPPH^•^ inhibition assays were 2.9 and 1.7 Trolox equivalent antioxidant capacity—TEAC/g, respectively [28].

The obtained antioxidant activity values are probably due to the synergistic interaction of individual phenolic compounds. Several authors have identified and/or quantified them in chia seeds in previous studies [10,14,21,29,30], as shown in Table 5. Since the results of the oil revealed low values for the TPC and antioxidant activity, it can be expected that the phenolic profile will be similar in the whole seeds and in the defatted cake. In another study with methanolic extracts of chia seeds, fiber flour, and oil, 14 phenolic acid derivatives were identified in the crude extract and 21 phenolic acids derivatives in the hydrolyzed extract, such as caffeic acid, danshensu, rosmarinic acid, methyl rosmarinic acid, salvianolic acid C, and salvianolic acid E [10]. The consumption of chia seeds can be an important alternative to improve consumer health, and the obtained results suggest its use as a functional food in the daily diet. However, the health advantages of eating these antioxidants should be studied using in vitro digestion models.

The beneficial effects of the consumption of bioactive compound will depend on the composition and modification during digestion. A study simulated the in vitro gastrointestinal digestion of non-defatted and defatted chia seeds [31]. There were no significant differences in the recovery index (RI) between both samples after digestion. However, the oral phase seems to negatively affect the TPC since the RI was lower in both samples in comparison to the reference matrix. Despite that, the highest RI results were obtained after the intestinal phase, pointing to a release of compounds from the matrix in the gastric phase, probably by hydrolysis. The bioaccessibility index (BI) was higher for the TPC than for the TFC in non-defatted chia but similar values were obtained for defatted chia. There were no differences observed in the TFC; nevertheless, the defatted sample had a lower RI, after the intestinal stage, than the non-defatted sample. There was also a decrease in the RI after the oral phase considering the reference matrix. The TFC was affected by the digestion process, since after digestion, the RI increased for the non-defatted sample similar to the reference matrix, but that did not happen for the defatted sample, which presented a similar RI to the gastric phase. This may be related to the low stability of flavonoids and their interaction with the food matrix compounds, particularly the fat content that could retain them. Defatted chia had similar BI values, whereas the BI for phenolics was higher than that for flavonoids for non-defatted chia. Overall, the TPC increased during digestion, but only low medium and low percentages of phenolics and flavonoids, respectively, are available for absorption in the intestinal tract. Moreover, the presence of high levels of fat seems to negatively impact on the bioaccessibility of flavonoids. The low bioaccessibility values were obtained for both phenolics and flavonoids; nevertheless, their potential antioxidant capacity supports the use as food ingredients of non-defatted and defatted chia seeds [31]. This suggests that the antioxidants could be more available after digestion in chia cake due to the lower fat content (Table 3).

In the Portuguese food market, to the best of our knowledge, there are still no foodstuffs containing chia defatted cake. Instead, there are several products containing whole seeds or chia flour, which corresponds to the entire ground seeds. Some examples of available products are energy bars, beverages, biscuits, whole-grain bread, breakfast mix, cookies, crackers, granola, margarine, muesli, oil, peanut butter, porridge, protein powder, pudding mix, dietary supplements, and yogurt.

In another study, cookies were produced using chia defatted cake. The partial or total substitution of wheat flour by the chia cake allowed the improvement of the nutritional characteristics of cookies, increasing the protein and fiber contents. However, the use of this gluten-free ingredient results in less cohesive cookies with a less elastic behavior. Thus, they are more brittle and prone to crumbling. Regarding the sensory analysis, the wheat cookies were preferred to chia cookies. In that study, brownies were also produced with this ingredient, a healthier product was obtained with lower contents of saturated fatty acids [32]. Mas et al., in 2020, also produced cookies using defatted chia flour (DCF) at 5, 10 and 20%. The addition did not affect the technological quality of cookies, except for color. Moreover, 10% DFC cookies were preferred over the others considering the sensorial analysis. The addition of DFC increased the polyphenol content and the in vitro antioxidant capacity. The simulated gastrointestinal digestion showed that 73% of total polyphenols could be absorbed in the intestine, also showing prebiotic effects [33]. Another study substituted 5 or 10% of wheat flour by several types of residual chia flour (whole, semi-defatted, and defatted, with or without mucilage) in bread formulations. The water absorption, dough development time, and stability of blends with mucilage as well as the incorporation of 10% of chia flour demonstrated the highest values. The specific volume of the flour variants with 5% of chia flour with mucilage were similar to the control but those formulated without mucilage exhibited a lower volume. The incorporation of 10% of chia flour in the formulations caused a decrease in the quality of the bread crust and crumb color. The incorporation of 5% of chia flour with mucilage improved the technological quality of the bread formulations. The different oil contents of chia samples did not show any significant influence on the overall quality or texture [34].

Overall, chia cake is a good option to be included in the food supply chain as an ingredient with high-fiber content and high-quality protein to formulate novel foodstuffs. Since heat can decompose natural antioxidants, in this case, raw formulations such as energy bars could be an interesting approach. For baked formulations, the stability of phytochemicals should be further researched. Thus, providing applications for chia cake will help to achieve a circular economy with a zero-waste approach and achieve sustainable diets [6].

## 3. Materials and Methods

### 3.1. Samples

Chia seeds were obtained from Peru and Bolivia. For oil extraction, 1 kg of seeds was subjected to cold-pressing using a screw press Komet Oil Press CA59G (IBG Monforts Oekotec GmbH & Co. KG, Monchengladbach, Germany), at room temperature, where oil and cake were obtained. The seeds and cake were vacuum-sealed and stored at 4 °C. The oil was stored in amber containers and underwent treatment with nitrogen stream to prevent oxidation. Before analysis, the seeds were ground in a mill (Retsch Knife Mill GRINDOMIX GM 200, Retsch, Haan, Germany).

### 3.2. Chemical Analysis of Cold-Pressed Chia Oil

#### 3.2.1. Oxidative Stability

The oxidative stability of chia oil was evaluated in a Rancimat apparatus (model 892, Metrohm Nordic ApS, Glostrup, Denmark). The tests were carried out with 3 g of oil, at 120 °C, and an airflow of 20 L/h, according to Melo et al., 2021 [17]. Results are expressed as the induction time (h).

#### 3.2.2. Color

The color of the oil was determined in a spectrophotometer (Shimadzu UV Spectrophotometer UV-1800, Kyoto, Japan), according to NP-937:1987 [35].

#### 3.2.3. Peroxide Value

The peroxide value of the oil was determined, according to NP-904:1987 [36], by mixing 0.5 g of oil, 10 mL of chloroform, 15 mL of glacial acetic acid, and 1 mL of saturated potassium iodide solution. The mixture was then stored in the dark for 5 min. After that, 75 mL of deionized water was added and mixed, followed by a titration with sodium thiosulphate (0.01 N) and starch solution (1%).

#### 3.2.4. UV Absorbance

The UV absorbance of the oil was determined in a spectrophotometer (Shimadzu UV Spectrophotometer UV-1800, Kyoto, Japan), according to ISO 3656:2002 [37].

#### 3.2.5. Phytochemical Compound Extraction and Analysis

The extraction of phytochemical compounds from oil was performed according to the method described by Capannesi et al., in 2000 [38], with 80/20% methanol/water (*v*/*v*).

##### Total Phenolic Compounds

The total phenolic compounds (TPC) in oil were determined according to the method described by Costa et al. in 2018 [39]. Briefly, 30 μL of the extract, 150 µL of Folin-Ciocalteu reagent (1:10), and 120 µL of sodium carbonate solution (7.5% m/V) were mixed and incubated (15 min, 45 °C). After 30 min, the absorbance was read at 765 nm in a microplate reader (Synergy HT GENS5, BioTek Instruments, Winooski, VT, USA). A calibration curve was prepared with the standard—gallic acid (5–100 mg/L).

##### Total Flavonoids Content

The total flavonoids content (TFC) of oil was determined according to the method described by Costa et al., in 2018 [39]. Briefly, 1 mL of the extract, 4 mL of deionized water, and 300 μL of sodium nitrite (25%) were mixed. After 5 min, 300 μL of aluminum chloride (10%) was added and mixed. After 1 min, 2 mL of sodium hydroxide (1 M) and 2.5 mL of deionized water were added and mixed. The absorbance was read at 510 nm in the same microplate reader as that used for determining the TPC. A calibration curve was prepared with the standard—epicatechin (2.5–400 mg/L).

##### Ferric Reducing Antioxidant Power

The ferric reducing antioxidant power (FRAP) of the oil was analyzed according to the method described by Costa et al. in 2018 [39]. Briefly, 30 μL of the extract was mixed with 270 µL of the FRAP solution (0.3 M acetate buffer; 10 mM 2,4,6-tris(2-pyridyl)-s-triazine—TPTZ solution; and 20 mM ferric chloride). The mixture was protected from light (30 min, 37 °C). The absorbance was read at 595 nm in the same microplate reader as that used for determining the TPC. A calibration curve was prepared with the standard—ferrous sulphate (25–500 mg/L).

##### 2,2-Diphenyl-1-Picrylhydrazyl Radical Inhibition

The 2,2-diphenyl-1-picrylhydrazyl radical (DPPH^•^) inhibition of the oil was analyzed according to the method described by Costa et al. in 2018 [39]. Briefly, 30 μL of the extract was mixed with 270 μL of freshly prepared DPPH^•^ solution (6 × 10^−5^ mol/L in ethanol). The absorbance at 525 nm was measured every 2 min to observe the kinetics reaction, with the endpoint at 20 min, in the same microplate reader as that used for determining the TPC. A calibration curve was prepared with the standard—Trolox (5.62–175.34 mg/L).

#### 3.2.6. Fatty Acids Profile

The fatty acid profile of the oil was determined via GC-FID (gas chromatography-flame ionization detector). A basic transmethylation was performed to obtain fatty acid methyl esters (FAMEs), according to ISO 12966-2:2017 [40].

The FAMEs were then injected in a GC-FID system (Shimadzu, Tokyo, Japan) coupled to an AOC-20i automatic sampler and a split/splitless auto injector at 250 °C and a flame ionization detector at 270 °C. A CP-Sil 88 silica capillary column (50 m × 0.25 mm, 0.2 μm, Varian, Middelburg, The Netherlands) was used for separation. The carrier gas and the injection volume were helium and 1 μL, respectively. The temperature program used for peaks separation was: 120 °C (5 min), 2 °C/min to 160 °C, 160 °C (2 min), 2 °C/min to 220 °C, and 220 °C (10 min). Identification was performed by comparing the FAME retention times with those of a standard mixture FAME 37 (Supelco, Bellefonte, PA, USA).

#### 3.2.7. Vitamin E Profile

The vitamin E profile of the oil was determined via HPLC-DAD-FLD (high performance liquid chromatography-diode array detector-fluorescence light detector). An appropriate amount of oil was mixed with 950 µL of *n*-hexane and 50 µL of the internal standard—tocol (100 μg/mL). The samples were then injected in a HPLC system (Jasco, Tokyo, Japan) coupled to a MD-4015 multiwavelength diode array detector used for the identification of isomers (α-tocopherol, β-tocopherol, γ-tocopherol, δ-tocopherol, α-tocotrienol, β-tocotrienol, γ-tocotrienol, and δ-tocotrienol), a FP-4025 fluorescence detector used for quantification (λ_exc_ = 290 nm, λ_em_ = 330 nm), a PV-4180 pump, an AS-4050 autosampler, and a Supelcosil TM LC-SI normal phase column used for separation (7.5 cm × 3 mm, 3 μm, Supelco, Bellefonte, PA, USA). The eluent, flow rate, and injection volume were 1.2% of 1,4-dioxane in *n*-hexane (*v*/*v*), 0.7 mL/min, and 20 μL, respectively.

### 3.3. Chemical Analysis of Chia Seeds and Cake

#### 3.3.1. Moisture Content

The moisture content of the chia cakes and seeds was determined in an infrared moisture analyzer (DBS—KERN & SOHN GmbH, Balingen, Germany) at 105 °C.

#### 3.3.2. Macro Composition

The macro composition of the chia seeds and cake was determined by using the AOAC methods described in 2019 [41]: the ash was quantified after incineration (AOAC 920.153); the total protein content was determined via the Kjeldahl method (AOAC 928.08)—6.25 was the nitrogen conversion factor [42]; the total fat content was determined using the Soxhlet method (AOAC 991.36); the dietary fiber and insoluble fiber were determined using the enzymatic-gravimetric procedures (AOAC 985.29 and AOAC 991.42, respectively); and soluble fiber and remaining carbohydrates were calculated using the difference [42].

#### 3.3.3. Energy Values

Energy values were calculated according to the European Commission Regulation [43]:

Energy value (kcal/100 g) = (g of protein × 4) + (g of fat × 9) + (g of carbohydrates × 4) + (g of fiber × 2)(1)

Energy value (kJ/100 g) = (g of protein × 17) + (g of fat × 37) + (g of carbohydrates × 17) + (g of fiber × 8)(2)

#### 3.3.4. Total Amino Acids

The total amino acids in the chia cakes and seeds were determined by using HPLC-DAD-FLD. The total amino acids were obtained via alkaline (KOH 4 M, 4 h, for tryptophan) and acid hydrolysis (HCl 6 M, 24 h, for the other amino acids). Aliquots of neutralized hydrolysates were mixed with the internal standard norvaline (2 mg/mL), according to the method described by Machado et al., in 2020 [44]. The mixtures were then injected in a HPLC system (Jasco, Tokyo, Japan) that was composed of a LC-NetII/ADC hardware interface; two Jasco PU-980 pumps; an AS-4150 RHPLC autosampler (operating with automatic online derivatization with the reagents OPA/3-MPA and FMOC); a MD-2015 Plus multiwavelength detector (that was used for identification); and a FP-2020 Plus fluorescence detector (that was used for quantification, at λ_exc_ = 340 nm and λ_em_ = 450 nm, from 0–26.2 min, for OPA-derivatives; and at λ_exc_ = 266 nm and λ_em_ = 305 nm, from 26.2–40 min, for FMOC-derivatives). A ZORBAX Eclipse Plus C18 column (4.6 × 250 mm, 5 μm, Agilent Technologies, Santa Clara, CA, USA) was used for separation in a gradient solvent system.

#### 3.3.5. Protein Quality

Protein quality was calculated according to the methods given by the WHO/FAO/UNU in 2007 [25] and Oser in 1959 [45]:

Amino acid chemical score: AACS (%) = (mg of AA in 1 g test protein/mg of AA in 1 g requirement protein) × 100(3)

(4) $$\mathrm{Essential}\;\mathrm{amino}\;\mathrm{acids}\;\mathrm{index}:\;\mathrm{EAAI}\;(\%)=n^{\log}{\;}^{\mathrm{EAA}},\;\mathrm{where}\;\log EAA=\frac{1}{n}\left(log\frac{100\;a1}{a1R}+\ldots +log\frac{100\;an}{anR}\right)$$

#### 3.3.6. Lipid Fraction Extraction and Analysis

The lipid fraction of the chia cakes and seeds was extracted according to the method given by Melo et al. in 2021 [17].

##### Fatty Acids Profile

The fatty acids in the chia cakes and seeds were derivatized to FAMEs via basic transmethylation, according to ISO 12966-2:2017 [40]. The fatty acid profiles were determined by using GC-FID, using the same conditions previously described for the oil.

##### Vitamin E Profile

The vitamin E profiles of the chia cakes and seeds were analyzed by using HPLC-DAD-FLD, using the same conditions previously reported for the oil.

#### 3.3.7. Phytochemicals Extraction and Analysis

The extraction of phytochemicals from the chia cakes and seeds was performed according to the method described by Melo et al. in 2021 [17], using 80/20% methanol/water (*v*/*v*) in agitation (1 h, 40 °C). 

The TPC, TFC, FRAP, and DPPH^•^ inhibition assays were analyzed using the same conditions previously described for the oil.

### 3.4. Statistical Analysis 

The independent-samples *t*-test was used to compare results from cake and seeds, and one-way ANOVA followed by the post-hoc Tukey’s test were used to compare fatty acid profiles and to assess significant differences between samples (*p* < 0.05) with IBM SPSS Statistics (version 26, IBM Corp., Armonk, NY, USA).

## 4. Conclusions

Chia oil should be consumed raw due to its low oxidative stability, being a dietary source of essential FA (ALA and LA) and vitamin E (α, γ, and δ-tocopherols). However, it may be restricted to an exclusive market, because of its high price. Chia oil can have further applications, for instance, as a dietary supplement. The oil extraction process via cold-pressing was efficient in removing the majority of the oil from the seed.

The cake has great potential for the formulation of functional foods as a high source of protein and mainly dietary fiber, as shown in the current study. The protein is gluten-free and can be consumed by patients with coeliac disease and can be an alternative to animal protein in food formulations. The evaluation of protein quality revealed that chia protein is balanced and of high quality. Furthermore, the consumption of fiber beneficially stimulates the intestinal microbiota known to promote health and well-being. 

Chia seeds combine all the above-mentioned nutritional features. In the current food market, the use of chia food products is increasing due to consumers’ search for natural foods with less industrial processing. It seems that if these ingredients are consumed raw, the potential health benefits of their phytochemical composition can be obtained.

## Figures and Tables

**Table 1:** Chemical analysis of cold-pressed chia oil and literature data.

Parameter	Results	Literature Data [Ref.]
Oxidative stability (h)	1.0 ± 0.0 (3 g, 120 °C, 20 L/h)	2.3 (5 g, 98 °C, 20 L/h) [7]; 2.57 (5 g, 100 °C, 20 L/h) [8]
Color (x, y)	(0.4223, 0.4365)	-
Transparency (%)	67.8	-
Dominant wavelength (nm)	576.6	-
Purity	62.2	-
K_232nm_	0.016 ± 0.001	1.35–1.47 [9]; 1.99 [8]
K_270nm_	0.0033 ± 0.0002	0.22–0.67 [9]; 0.46 [8]
Peroxide value (meq O_2_/kg)	2.4 ± 0.0	0.14–2.67 [9]; 0.45 [8]
TPC (mg GAE/100 g)	1.3 ± 0.0	2 mg GAE/100 g [10]
TFC (mg ECE/100 g)	ND	-
FRAP (μmol FSE/100 g)	35.4 ± 3.2	26 µmol TE/100 g [10]
DPPH^•^ inhibition (mg TE/100 g)	ND	-
α-Tocopherol (mg/kg)	4.9 ± 0.2	8 [11]; 0.4–9.9 [12]; traces [8]
γ-Tocopherol (mg/kg)	389.0 ± 2.1	422 [11]; 330 [8]
δ-Tocopherol (mg/kg)	14.7 ± 0.9	15 [11]; 14 [8]
Total vitamin E (mg/kg)	408.5 ± 0.3	445 [11]; 238–427 [12]; 344 [8]

Results are presented as mean ± standard deviation (*n* = 3). (x, y)—chromatic coordinates, K—extinction coefficient, TPC—total phenolic compounds, GAE—gallic acid equivalents, TFC—total flavonoids content, ECE—epicatechin equivalents, FRAP—ferric reduction antioxidant power, FSE—ferrous sulphate equivalents, DPPH^•^—2,2-diphenyl-1-picrylhydrazyl radical inhibition, TE—Trolox equivalents, ND—not detected.

**Table 2:** Fatty acids profile of chia seeds, cake, oil, and literature data.

Fatty Acids	Seeds	Cake	Oil	Seeds	Seeds	Oil	Oil	Oil
Reference	-	-	-	[11]	[14]	[13]	[15]	[8]
C14:0	-	-	-	0.06	0.03	0.07	-	-
C15:0	-	-	-	0.04	0.03	0.05	-	-
C16:0	6.62 ± 0.11 b	7.67 ± 0.09 a	6.39 ± 0.11 b	7.10	6.69	7.07	6.21	7.03
C16:1	0.14 ± 0.01 a	0.15 ± 0.00 a	0.10 ± 0.01 b	0.20	0.09	0.08	-	0.04
C17:0	0.05 ± 0.01 a	0.06 ± 0.00 a	-	0.06	0.06	0.07	-	-
C17:1	-	-	-	0.06	-	0.03	-	-
C18:0	2.63 ± 0.03 b	3.51 ± 0.03 a	2.46 ± 0.17 b	3.24	2.67	3.36	1.89	3.27
C18:1n9c	5.42 ± 0.09 c	5.66 ± 0.02 b	6.19 ± 0.09 a	10.53	10.55	7.04	5.68	7.46
C18:2n6c	18.83 ± 0.17 b	20.24 ± 0.11 a	18.82 ± 0.05 b	20.37	17.36	18.23	21.46	19.71
C18:3n3c	65.96 ± 0.37 a	62.15 ± 0.23 b	65.98 ± 0.13 a	59.76	62.02	62.80	64.39	60.72
C20:0	0.25 ± 0.01 b	0.33 ± 0.01 a	-	0.24	-	0.29	-	-
C20:1n9	-	-	-	0.16	0.09	0.14	-	-
C20:2	-	-	-	0.07	0.03	-	-	-
C22:0	-	-	-	0.08	0.09	0.09	-	-
C24:0	0.11 ± 0.01 b	0.24 ± 0.02 a	0.07 ± 0.07 c	0.10	0.14	0.12	-	-
∑SFA	9.65 ± 0.14 b	11.80 ± 0.13 a	8.91 ± 0.15 c	8.65	9.74	11.12	8.50	12.07
∑MUFA	5.57 ± 0.09 c	5.81 ± 0.02 b	6.29 ± 0.09 a	10.95	10.76	7.29	5.68	7.50
∑PUFA	84.78 ± 0.22 a	82.38 ± 0.12 b	84.80 ± 0.11 a	80.40	79.47	81.59	85.85	80.43
18:2n6/18:3n3	0.29 ± 0.00 b	0.33 ± 0.00 a	0.29 ± 0.00 b	0.35	0.28	0.29	0.33	0.32
18:1n9/18:2n6	0.29 ± 0.00 b	0.28 ± 0.00 b	0.33 ± 0.00 a	0.52	0.61	0.39	0.26	0.38

Results are presented as the relative percentage of total fatty acids (%). Values are presented as the mean ± standard deviation (*n* = 3). Different letters in the same row denote significant differences between samples (*p* < 0.05) on the one-way ANOVA followed by the post-hoc Tukey’s test. C14:0—myristic acid, C15:0—pentadecanoic acid, C16:0—palmitic acid, C16:1—palmitoleic acid, C17:0—margaric acid, C17:1—cis-10-heptadecenoic acid, C18:0—stearic acid, C18:1n9c—oleic acid, C18:2n6c—linoleic acid, C18:3n3c—α-linolenic acid, C20:0—arachidic acid, C20:1n9—eicosanoic acid, C20:2—cis-eicosadienoic acid, C22:0—behenic acid, C24:0—lignoceric acid, SFA—saturated fatty acids, MUFA—monounsaturated fatty acids, PUFA—polyunsaturated fatty acids.

**Table 3:** Chemical analysis of chia seeds, cake, and literature data.

Parameter	Seeds	Cake	Seeds [Ref.]	Defatted Flour [Ref.]
Energy value (kJ/100 g)	1827 a	1173 b	-	-
Energy value (kcal/100 g)	444 a	284 b	-	-
Moisture (%)	6.57 ± 0.03 b	7.02 ± 0.20 a	5.6–7.1 [18]; 3.3 [19]	-
Ash (%)	4.67 ± 0.02 b	6.42 ± 0.04 a	4.6–5.1 [18]; 5.5 [19]; 5.1 DW [20]	7.7 DW [20]
Total protein (%)	18.08 ± 0.41 b	26.66 ± 0.09 a	18.2–19.7 [18]; 23.2 [19]; 21.4 DW [20]	32.0 DW [20]
Total fat (%)	32.85 ± 0.17 a	6.73 ± 0.43 b	30.2–32.2 [18]; 28.4 [19]; 33.7 DW [20]	8.8 DW [20]
Total dietary fiber (%)	37.71 ± 0.00 b	48.11 ± 3.68 a	33.4–37.2 [18]; 37.4 [19]; 25.6 DW [20]	29.2 DW [20]
Insoluble fiber (%)	35.20 ± 0.00 b	39.37 ± 0.61 a	-	-
Soluble fiber (%)	2.51 ± 0.00 b	8.74 ± 2.17 a	-	-
Remaining carbohydrates (%)	0.12 ± 0.18 b	5.06 ± 2.39 a	-	-
Aspartic acid (mg/g)	17.64 ± 0.61 b	24.10 ± 0.42 a	-	-
Glutamic acid (mg/g)	36.15 ± 1.53 b	48.93 ± 0.46 a	-	-
Serine (mg/g)	12.02 ± 0.36 b	16.07 ± 0.12 a	-	-
Glutamine (mg/g)	2.50 ± 0.67 b	4.63 ± 0.12 a	-	-
^1^ Histidine (mg/g)	8.17 ± 0.23 b	10.67 ± 0.17 a	-	-
Glycine (mg/g)	11.25 ± 1.24 b	14.26 ± 0.17 a	-	-
^1^ Threonine (mg/g)	7.58 ± 0.23 b	10.03 ± 0.07 a	-	-
Arginine (mg/g)	25.86 ± 1.12 b	33.82 ± 0.35 a	-	-
Alanine (mg/g)	10.39 ± 0.41 b	13.82 ± 0.17 a	-	-
Tyrosine (mg/g)	5.09 ± 0.37 b	5.93 ± 0.06 a	-	-
^1^ Valine (mg/g)	8.85 ± 0.24 b	11.78 ± 0.23 a	-	-
^1^ Methionine (mg/g)	3.68 ± 0.52 a	3.38 ± 0.22 a	-	-
^1^ Tryptophan (mg/g)	1.38 ± 0.01 a	1.34 ± 0.19 a	-	-
^1^ Phenylalanine (mg/g)	10.65 ± 0.45 b	14.06 ± 0.26 a	-	-
^1^ Isoleucine (mg/g)	7.31 ± 0.28 b	9.61 ± 0.26 a	-	-
^1^ Leucine (mg/g)	13.61 ± 0.51 b	18.21 ± 0.25 a	-	-
^1^ Lysine (mg/g)	14.22 ± 2.71 a	17.31 ± 0.40 a	-	-
Hydroxyproline (mg/g)	0.75 ± 0.03 b	1.08 ± 0.01 a	-	-
Proline (mg/g)	6.24 ± 0.84 b	9.48 ± 0.15 a	-	-
∑Branched-chain amino acids (mg/g)	29.76 ± 1.00 b	39.60 ± 0.68 a	-	-
∑Essential amino acids (mg/g)	74.07 ± 2.11 b	96.39 ± 1.45 a	-	-
∑Total amino acids (mg/g)	203.33 ± 4.29 b	268.51 ± 3.13 a	-	-
α-Tocopherol (mg/kg)	59.25 ± 0.83 a	14.03 ± 0.23 b	-	-
γ-Tocopherol (mg/kg)	137.42 ± 3.25 a	29.44 ± 0.40 b	-	-
δ-Tocopherol (mg/kg)	3.48 ± 0.16 a	0.92 ± 0.06 b	-	-
∑Total vitamin E (mg/kg)	200.16 ± 3.79 a	44.38 ± 0.65 b	82 mg/kg [18]	
TPC (mg GAE/100 g)	187.5 ± 9.1 a	199.2 ± 12.9 a	1.2 mg GAE/g [10]; 88 mg/100 g [21]; 64 mg/100 g [14]; 2.6 g of GAE/kg DW [19]	1.1 mg GAE/g [10]
TFC (mg ECE/100 g)	221.0 ± 17.3 b	309.3 ± 6.8 a		-
FRAP (mmol FSE/100 g)	11.1 ± 0.3 b	15.2 ± 0.9 a	74 mol TE/g [10]; 45 mmol TE/kg DW [19]	69 mol TE/g [10]
DPPH^•^ (mg TE/100 g)	129.9 ± 21.1 a	144.2 ± 22.9 a	-	-

^1^ Essential amino acids. Results are presented as mean ± standard deviation (*n* = 3) in fresh weight. Different letters in the same row denote significant differences (*p* < 0.05) on the independent-samples *t*-test. DW—dry weight, TPC—total phenolic compounds, GAE—gallic acid equivalents, TFC—total flavonoids content, ECE—epicatechin equivalents, FRAP—ferric reduction antioxidant power, FSE—ferrous sulphate equivalents, DPPH^•^—2,2-diphenyl-1-picrylhydrazyl, TE—Trolox equivalents.

**Table 4:** Protein quality of chia seeds and cake.

EAA	AAs Estimates for Adults [25]	Seeds	Cake	Seeds AACS	Cake AACS
Units	mg/g protein	mg/g protein	mg/g protein	%	%
His	15	45.21 ± 1.26 a	40.02 ± 0.63 b	301.39 ± 8.41 A	266.79 ± 4.22 B
Ile	30	40.43 ± 1.54 a	36.06 ± 0.98 b	134.75 ± 5.14 A	120.20 ± 3.27 B
Leu	59	75.26 ± 2.79 a	68.29 ± 0.93 b	127.56 ± 4.74 A	115.75 ± 1.58 B
Lys	45	78.66 ± 15.01 a	64.92 ± 1.52 b	174.80 ± 33.37 A	144.26 ± 3.37 B
Met	16	20.36 ± 2.85 a	12.67 ± 0.81 b	127.28 ± 17.83 A	79.21 ± 5.08 B
Phe + Tyr	38	87.05 ± 4.49 a	74.96 ± 1.17 b	229.07 ± 11.82 A	197.26 ± 3.08 B
Tre	23	41.93 ± 1.29 a	37.64 ± 0.27 b	182.30 ± 5.61 A	163.64 ± 1.15 B
Trp	6	7.61 ± 0.03 a	5.02 ± 0.72 b	126.80 ± 0.56 A	83.72 ± 12.04 B
Val	39	48.94 ± 1.32 a	44.19 ± 0.85 b	125.49 ± 3.40 A	113.30 ± 2.17 B
LAA (%)	-	-	-	Val 125.49 ± 3.40 A	Met 79.21 ± 5.08 B
EAAI (%)	-	128.05 ± 2.34 a	106.21 ± 1.57 b	-	-

Results are presented as mean ± standard deviation (*n* = 3). AAs—amino acids, AACS—amino acid chemical score, EAA—essential amino acid, LAA—limiting amino acid, EAAI—essential amino acid index. Different small letters in the same row denote significant differences between the results for the seeds and cake in mg/g protein on the independent-samples *t*-test (*p* < 0.05). Different capital letters in the same row denote significant differences between the AACS results of the seeds and cake on the independent-samples *t*-test (*p* < 0.05).

**Table 5:** Phenolic compounds of chia seeds.

Compound	Quantity	Origin	Reference
Caffeic acid	-	Chile	[10]
	0.030 mg/g	Brazil	[14]
	0.003–0.006 mg/g	Mexico	[21]
	0.0274 mg/g	Mexico	[29]
	0.139–0.149 mg/g	Ecuador	[30]
Ferulic acid	-	Chile	[10]
	Traces	Mexico	[29]
Chlorogenic acid	0.004 mg/g	Brazil	[14]
	0.102–0.045 mg/g	Mexico	[21]
	0.226–0.218 mg/g	Ecuador	[30]
Rosmarinic acid	-	Chile	[10]
	0.9267 mg/g	Mexico	[29]
Myricetin	0.115–0.121 mg/g	Ecuador	[30]
Quercetin	0.17 µg/g	Brazil	[14]
	0.150–0.268 mg/g	Mexico	[21]
	0.007–0.006 mg/g	Ecuador	[30]
Kaempferol	0.360–0.509 mg/g	Mexico	[21]
	0.025–0.024 mg/g	Ecuador	[30]
Daidzin	0.006 mg/g	Mexico	[29]
Glycitein	0.0005 mg/g	Mexico	[29]
Glycitin	0.0014 mg/g	Mexico	[29]
Genistein	0.0051 mg/g	Mexico	[29]
Genistin	0.0034 mg/g	Mexico	[29]

## Data Availability

Not applicable.
